# A Clinical Decision Support System for Remote Monitoring of Cardiovascular Disease Patients: A Clinical Study Protocol

**DOI:** 10.3389/fpubh.2022.859890

**Published:** 2022-05-09

**Authors:** Filipa Ventura, Pedro Sousa, Maria Anjos Dixe, Paulo Ferreira, Ricardo Martinho, Sara Simões Dias, João Morais, Lino M. Gonçalves

**Affiliations:** ^1^The Health Sciences Research Unit: Nursing (UICISA: E), Nursing School of Coimbra (ESEnfC), Coimbra, Portugal; ^2^Center for Innovative Care and Health Technology (ciTechcare), School of Health Sciences, Polytechnic of Leiria, Leiria, Portugal; ^3^School of Technology and Management, Polytechnic of Leiria, Leiria, Portugal; ^4^CINTESIS-Center for Health Technology and Services Research, University of Porto, Porto, Portugal; ^5^Cardiology Division, Leiria Hospital Center, Leiria, Portugal; ^6^Cardiology Department, Coimbra University Hospital Centre, Coimbra, Portugal; ^7^Faculty of Medicine, University of Coimbra, Coimbra, Portugal

**Keywords:** mHealth, cardiac rehabilitation, self-management, personalized care, clinical study

## Abstract

**Introduction:**

Cardiovascular diseases (CVD) are the leading cause of death globally, taking an estimated 17. 9 million lives each year. Cardiac rehabilitation is shown to reduce mortality and hospital readmissions, while improving physical fitness and quality of life. Despite the recommendations and proven benefits, acceptance and adherence remain low. Mobile health (mHealth) solutions may contribute to more personalized and tailored patient recommendations according to their specific needs. This study protocol aims to assess the effectiveness of a user-friendly, comprehensive Clinical Decision Support System (CDSS) for remote patient monitoring of CVD patients, primarily on the reduction of recurrent cardiovascular events.

**Methods and Analysis:**

The study will follow a multicenter randomized controlled design involving two cardiology units in the Center Region of Portugal. Prospective CVD patients will be approached by the healthcare staff at each unit and checked for eligibility according to the predefined inclusion/exclusion criteria. The CDSS will suggest a monitoring plan for the patient, will advise the mHealth tools (apps and wearables) adapted to patient needs, and will collect data. The clinical study will start in January 2023.

**Discussion:**

The success of the mHeart.4U intervention will be a step toward the use of technological interfaces as an integrating part of CR programs.

**Ethics and Dissemination:**

The study will undergo ethical revision by the Ethics Board of the two hospital units where the study will unfold. The study was registered in ClinicalTrials.gov on 18th January 2022 with the number NCT05196802. The study findings will be published in international peer-reviewed scientific journals and encounters and in a user-friendly manner to the society.

## Introduction

Cardiovascular diseases (CVD) are the most common non-communicable diseases globally, accounting for an estimated 17.9 million deaths per year (1). They are the leading cause of mortality worldwide, with the number of deaths increasing by 14.5% between 2006 and 2016 (2). The reduction of CVD-related mortality and morbidity is a key global health priority for the World Health Organization (WHO) (3) and the United Nations (UN) Sustainable Development Goals (4).

The American Heart Association and the European Society of Cardiology suggest that cardiac rehabilitation (CR) is a Class IA recommendation for patients with CVD. Substantial benefits include reducing mortality by 20 to 47%, reducing hospital readmissions by 18%, improving physical activity, reducing cardiovascular risk factors and improving quality of life (5, 6). Despite the proven benefits, low rates of adherence (14 to 35.5%) in traditional CR programs continue to limit treatment impact, due to inadequate access, time conflicts and associated costs (5, 7).

Rapid advances in mobile wireless communications and wearable sensor technologies may bridge the gap between home- and center-based delivery models by combining availability, accessibility, and responsive individualized clinical oversight (8). These technologies have the potential to overcome some of these barriers and may be a valuable instrument for promoting adherence. Mobile technologies are recognized for benefits such as bridging time and distance barriers to clinical oversight and increasing accessibility to care that is traditionally delivered face-to-face (9).

Particularly in relation to CR, studies reveal that technology-mediated interventions are equally effective in improving health outcomes compared to conventional care, dissipating fears referred by both clinicians and patients in relation to achieving similar results with virtual methods (10, 11). Studies specifically evaluating the effectiveness of mHealth-mediated CR interventions have revealed positive impact on composite and combined scores involving cardiovascular morbidity (e.g., worsening heart failure), hospitalizations or readmissions due to cardiovascular causes (e.g., unplanned revascularization) and cardiovascular mortality (12).

The COVID-19 era has been of enormous importance for the clinical implementation of digital health and wearable devices (13). Due to this pandemic, outpatient visits of chronic patients have been replaced by virtual visits to limit disease transmission. Health-related mobile apps (mHealth) have many advantages such as enabling continuous monitoring of patient health status, receiving health-related knowledge and automated feedback, and improving quality of life (14–17). Studies have shown that the use of mHealth interventions increases motivation and participation in CR programs (8, 18). In 2017, there were an estimated 3.7 billion mHealth app downloads (19), highlighting their practicality and convenience.

However, there is still a lack of qualitative data about which mobile app features are more engaging over time, thus contributing to behavior change and increasing treatment adherence (1). The heterogeneity of features in these apps leads to the need of identifying which of them can better contribute to disease self-management (20).

Wearables may also provide a benefit through increased health awareness, democratization of health data and patient engagement (13). The widespread use of heart rate and fitness tracking technologies provides unparalleled opportunities for capturing physiological information. While the number of patients meeting healthcare providers with wearables is rapidly growing, the European Society of Cardiology (13) highlights that there are few clinical guidelines on how to use these data and that technical aspects of heart rate tracking need to be further validated. The use of continuous monitoring may allow early risk detection, thereby becoming novel applications in both prevention and clinical research (13). A recent systematic review (5) evaluated the effects of eHealth CR on health outcomes, showing a significant promotion of physical activity, daily steps, quality of life (QoL) and a reduction in rehospitalization.

Time constraints, patient overpopulation, and complex guidelines require alternative solutions for real-time patient monitoring. Rapidly evolving e-health technology combined with clinical decision support systems (CDSS) provides an effective solution to these problems (21). There are several computerized CDSS for chronic diseases management, however, to the best of our knowledge, there are none for the management of CVD patients.

Scoping the literature on remote monitoring of cardiovascular patients through mHealth tools, a few clinical study protocols can be found in recent years, aiming at determining the effect of these resources on both clinical (e.g., cardiovascular events) and process outcomes (e.g., hospital readmissions) (22, 23). Although overlapping the effect aimed through the mHeart.4U, the current study protocol adds to the previous as it builds on existing mHeath tools for monitoring and rehabilitation of cardiovascular patients with the integration of AI for personalized selection of the rehabilitation plan. Accordingly, the mHeart.4U trial will aim to reinforce the evidence on outcomes that inform clinical decision-making, such as rehospitalizations and health-related QoL, while following a sustainable approach to intervention research, wherein existing evidence and resources are pragmatically adapted and adopted to fit the real-world practice (24).

The current study protocol will innovatively explore CDSS for management of CVD patients building on existing mHealth-based interventions for cardiac monitoring and rehabilitation. We hypothesize that an mHealth intervention supported by a CDSS will reduce recurrent cardiovascular events, will promote QoL, treatment adherence, adoption of a healthier lifestyle, and Body Mass Index (BMI) reduction. Therefore, the objective of the mHeart.4U clinical study will be to determine the effectiveness of an intervention using a CDSS for remote patient monitoring, selecting the best mHealth tools (apps and wearables) according to the needs of each CVD patient and managing clinical data.

## Methods and Analysis

The mHeart.4U trial is designed in accordance with the methodology adopted for mHealth-based interventions for remote monitoring and rehabilitation trials involving CVD patients (5). Accordingly, the study will follow a pragmatic, multicenter, two parallel arms (1:1), prospective, randomized controlled design with blinded endpoint assessment, involving two cardiology units in the Center Region of Portugal. This study is a pragmatic trial because it examines the outcomes of the experimental intervention compared with a standard intervention under circumstances which closely approximate the real world (25). Additionally, personnel undertaking outcome assessment will be blinded to group allocation. The Standard Protocol Items: Recommendations for Interventional Trials reporting guidelines were used to write this protocol (26).

### Participants

Eligible participants will be adults (18+ years old) attending these cardiology outpatient clinics after the onset of an acute cardiac event, or those engaged in a structured CR program. Inclusion criteria also consider the need to be able to communicate with the researcher. Participants will be excluded if they have New York Heart Association class III/IV heart failure, terminal disease, or significant non-CVD exercise limitations.

### Procedures

**Figure 1:**
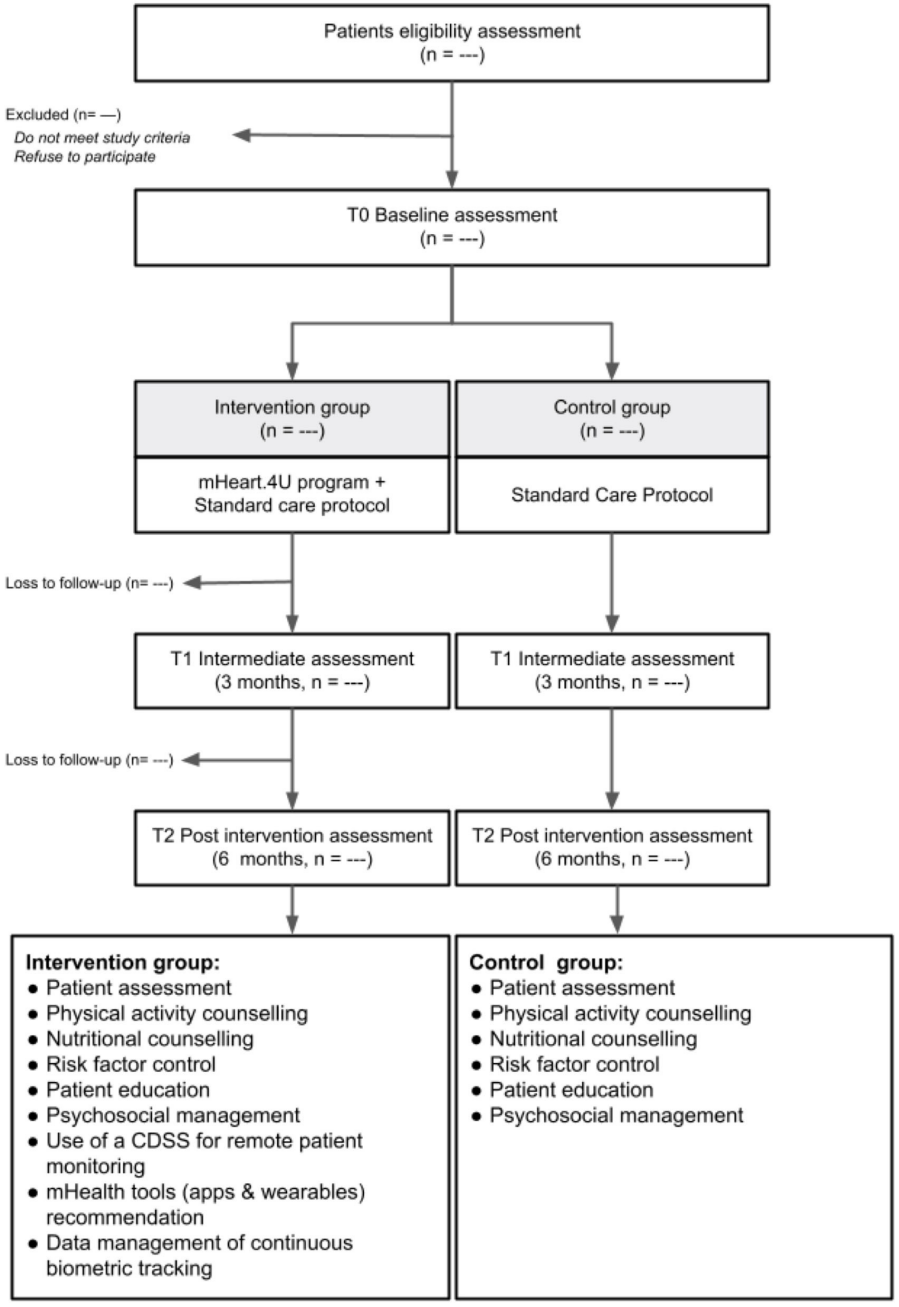
Participant's flowchart.

Sample recruitment will have the support of the clinical staff of the outpatient clinics based on inclusion and exclusion criteria. All eligible participants will be asked to fill out the informed consent. Participants will be randomized at a 1:1 ratio to receive usual care composed by CR alone (control group) or the mHeart.4U program (intervention group). Treatment allocation will follow a computer-generated schedule prepared by a biostatistician. The randomization schedule will be stratified by gender and trial center and will use random permuted blocks (Figure 1). The control group will follow the standard treatment protocol (27), while the Intervention group will, additionally, participate in the mHEART.4U intervention (Figure 1).

### Intervention

The mHEART.4U intervention will include the use of an online CDSS for remote patient monitoring. The CDSS rule sets will be developed according to the knowledge on: (a) self-management and monitoring needs and difficulties of CVD patients and requirements of healthcare professionals, and (b) functional and technical characteristics of available mHealth tools for promoting CR.

**Figure 2:**
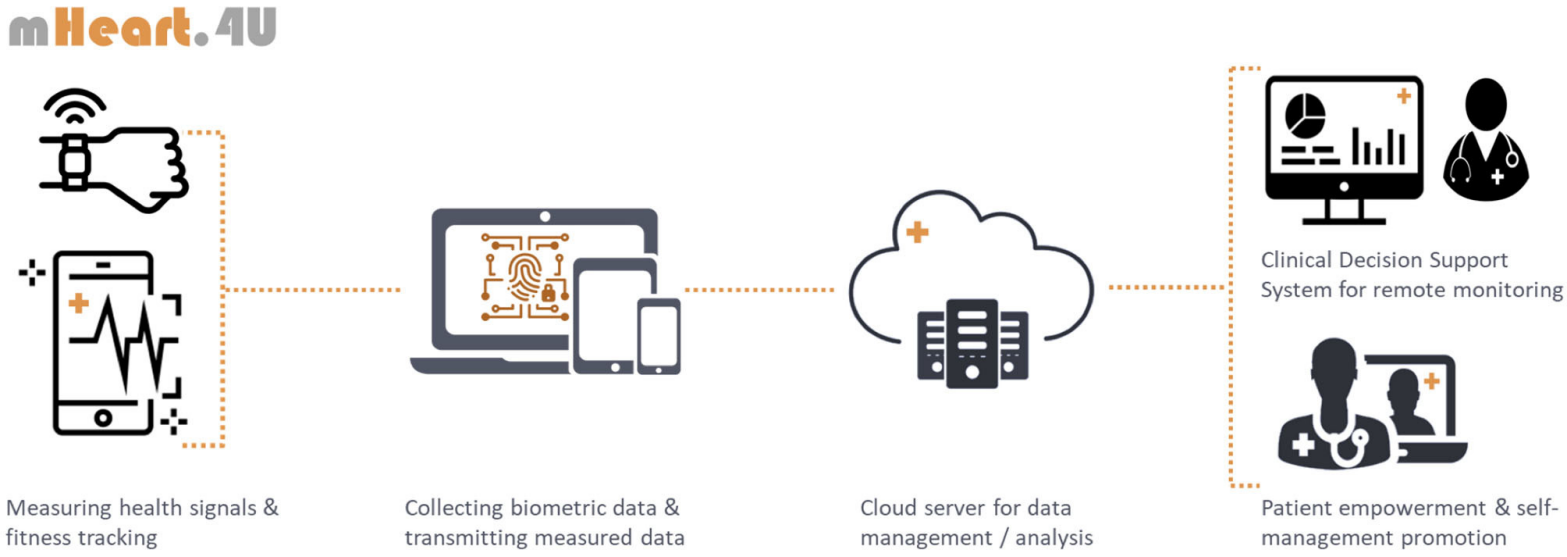
Overview of the mHeart.4U framework and functioning.

According to the patient's needs and profile, the CDSS will suggest a monitoring plan accordingly. The mHEART.4U intervention kit will include mobile apps and wearables, such as heart rate, blood pressure, peripheral oxygen saturation (SpO2), sleep and step trackers, symptoms, lifestyle self-monitoring tools, medication reminders or motivational resources. The resources composing each intervention kit will be defined upon initial assessment of the patient's needs and in a shared decision-making process according to co-established therapeutic goals (Figure 2). Smartphones and/or wearables may be lent if necessary.

Intervention length will be 6 months and will take into account the most recent guidelines on CR (27, 28).

### Outcomes and Measurements

Outcome measurements will be carried out at the 3-month (T1) and the 6-month interventions (T2) (Table 1).

**Table 1:** Schedule of assessments.

**Assessments**	**Measure**	**T0**	**T1**	**T2**
Recurrent cardiovascular event				
Cardiovascular	Patient health records		x	x
Rehospitalization	Death certificates			
Cardiovascular urgent visit				
Unplanned revascularization				
Cardiovascular mortality				
Worsening heart failure				
Quality of Life	MacNew heart disease health-related Quality of life questionnaire	x	x	x
Adherence to treatment	Therapeutic self-care scale	x	x	x
Anthropometric measures	Patient health records (BMI, waist circumference)	x	x	x
Nutrition	Health-promoting lifestyle profile-II	x	x	x
Physical activity	Health-promoting lifestyle profile-II	x	x	x
Clinical data	Patient health records	x	x	x
Blood pressure, heart rate,	Systematic coronary risk evaluation			
blood biomarkers, pathological				
history, cardiovascular risk				
Sociodemographic Data	Sociodemographic questionnaire	x		
Gender, age, academic				
qualifications, profession				
mHEART.4U usage	Utilization rate, consulted resources, self-monitoring data		x	x

*T0, Baseline assessment; T1, 3-month intervention assessment; T2, 6-month intervention assessment*.

The primary outcome will be the reduction of recurrent cardiovascular events, which is a composite of (i) cardiovascular rehospitalization or urgent visit; or (ii) unplanned revascularization; (iii) cardiovascular mortality; or (iv) worsening heart failure (29, 30).

Secondary outcomes measures will include:

a) Quality of Life, assessed through the MacNew Heart Disease Health-related Quality of Life (HRQL) questionnaire (MacNew) (31).

Acknowledging the importance of including patient's perspectives on health outcomes, the MacNew is a self-reported questionnaire that has been validated and used with both patients with experience of a myocardial infarction, and patients with experience of angina. The measurement instrument is composed of 27 items, scored from 1 (poor) to 7 (high), to assess global HRQL, physical limitations, emotional and social functions. The MacNew is currently validated to many languages including Portuguese, where it appears to be a reliable, valid and moderately responsive instrument to evaluate HRQL of people after a diagnosis of acute coronary syndrome (32);

b) Adherence to treatment, assessed through the Therapeutic Self-care Scale (TSC) (33).

The TSC includes 12 items to be answered on a 5-point Likert scale, from 0 (no) to 5 (yes) in relation to the level of knowledge during situations related to therapeutic self-care management, with higher scores corresponding to high level performance in therapeutic self-care. The measurement instrument is composed of four domains to assess patients' ability to engage in self-care activities related to: (1) taking medications as prescribed by the doctor; (2) identifying and managing symptoms; (3) performing activities of daily living; and (4) managing changes in condition. The instrument can be administered either to patients admitted to hospital with a variety of acute medical and surgical conditions, or to home-based care patients. The TSC has been widely used and is validated to various cultural contexts, including Portugal (34). The original scale has a strong internal consistency (Cronbach's α 0.93), also replicated in the Portuguese version (Cronbach's α 0.979);

c) Nutrition and Physical Activity, assessed through the Health-Promoting Lifestyle Profile-II (HPLP-II) (35).

The HPLP-II is a self-reporting instrument composed of 52 items to be answered on 4-point Likert scale (never, sometimes, often, routinely) in order to assess health-promoting behaviors as a multidimensional pattern of self-initiated actions and perceptions that serve to maintain or increase the level of wellbeing, self-fulfillment, and self-satisfaction. Nutrition (N, nine items) and Physical Activity (PA, eight items) are two of the six domains assessed, the others being: health responsibility (HR), spiritual growth (SG), interpersonal relations (IR), and stress management (SM). The original measurement instrument was tested with an α coefficient of 0.943. It has been used and validated to several languages and cultural contexts, including Portugal with psychometric results showing an adequate fit to a 52-item, six-factor structure and global Cronbach's α of 0.925 (α of N = 0.726; α of PA = 0.835) (36);

d) Anthropometric measures (Body Mass Index, waist circumference).

Other measures used during the randomized controlled trial include:

a) Sociodemographic characterization, gender, age, academic qualifications, and profession, which will be collected through questionnaires;
b) Clinical data, to be collected through questionnaires and patient records analysis, blood pressure, blood biomarkers, pathological history, and cardiovascular risk assessed through the Systematic COronary Risk Evaluation (SCORE) (37).

### Sample Size Calculation

The sample size calculation for the multicentre clinical trial will be performed in order to allow for a statistically significant comparison between control and intervention groups at each center with a 95% confidence level, two-tailed analysis and aiming at a reduction in the effect variable, i.e., recurrent cardiovascular events, which is a clinically relevant composite of (i) cardiovascular rehospitalization or urgent visit; or (ii) unplanned revascularization; (iii) cardiovascular mortality; or (iv) worsening heart failure (29, 30). The G Power software was used to determine *a priori* sample size according to the evidence reported in a previous systematic review (5), which showed that eHealth CR was effective reducing rehospitalization [RR = 0.49, 95% CI (0.27, 0.89), *p* = 0.02]. Accordingly, a proportion of 6% in the intervention group was rehospitalized compared with 13% in the control group 1 year after the intervention terminus, resulting in an effect size of 7% (5). Departing from this evidence-based measure of effect size, power (1 -β) of 80%, a significance of 0.05 and accounting for 20% loss to follow-up, a sample of 330 patients per arm will be included.

### Statistical Analysis

The patient characteristics in the two arms at baseline will be compared descriptively using chi-squared tests for the binary and categorical variables and an unpaired Student's *t*-test for the continuous variables. One-sample *t*-test will be performed to measure the differences within the same group.

The primary outcome will be analyzed using a generalized linear mixed model, adjusting for the stratification variables (i.e., gender, hospital unit). Logistic regression will be conducted in order to explore predictive relationships of the baseline categorical variables (e.g., age, clinical variables) on the primary outcomes in both groups.

Analyses to determine the study primary outcome will be performed on the principle of intention-to-treat. Accordingly, assuming there will be a reasonable amount (>5%) of missing data, analyses to determine the primary outcome will be conducted using a multiple imputation model. A per-protocol analysis will be conducted with complete cases to test the robustness of intervention effects under different assumptions. Subgroup analyses will be conducted to determine intervention effects by gender and trial center. All statistical tests will be 2-sided at α = 0.05. The biostatistician will remain blinded throughout the analysis of treatment effects.

## Discussion

The current study protocol aims to assess the effectiveness of a user-friendly, comprehensive CDSS for remote patient monitoring of CVD patients, primarily on the reduction of recurrent cardiovascular events. To that end a multicentre randomized controlled design involving two cardiology units in the Center Region of Portugal will be conducted involving patients living with CVD and receiving care at these Units. The CDSS will recommend a personalized monitoring plan for each patient through mHealth tools, and provide self-management recommendations adequate to the patient's needs and preferences.

Along with the WHO key global health priority (3) and UN Sustainable Development Goals (4) of reducing CVD-related mortality and morbidity, the mHeart.4U has the potential to contribute to sustainable and person-centerd CR, with low-effort added to the healthcare services and without jeopardizing quality of care (38). The mHeart.4U will be conducted at two cardiology units, which will allow to gain insight concerning contextual differences to the adoption of the intervention. The recruitment and intervention delivery might however entail minor differences that need to be investigated in a *post-hoc* analysis for their potential to induce bias.

Technology-mediated CR has been shown to have equal impact on health outcomes as conventional programmes delivered at the clinic (11). The mHeart.4U trial will primarily study the impact on recurrence of cardiac events, with effects on quality of life being secondarily explored. Along with the increasing importance of meaningfulness of life as a quality indicator for people living with chronic disease, recent trials have had the contrary approach [e.g., (23)]. Although it might be seen as a limitation, the evidence concerning this primary outcome is of paramount importance to ascertain the added value of the mHeart.4U in relation to the existing mHealth-based interventions for cardiac monitoring and rehabilitation, which will integrate the current trial. However, the mHeart.4U trial design does not allow for analyzing the separate effects of different mHealth resources included in the intervention.

Drop-out rates are recognizably higher in intervention studies involving mHealth resources (10, 15) and the mHeart.4U trial is not immune to that potential limitation. Attempting to mitigate the risk, evidence highlights the importance of attending to psychosocial variables that are related to adherence to mHealth interventions, and contextual factors related to adoption by healthcare professionals (39). As the mHeart.4U will entail personalized recommendations of mHealth resources complementary to standard-of-care, to be used according to the patients' preferences on daily living, the adherence to the intervention is expected to be reinforced.

To the patient participating in technology-based CR, the mHeart.4U is likely to empower to take action while recognizing his/her own resources beyond the disease (23). The patient will naturally become part of the therapeutic partnership in a more equitable role as the healthcare professionals, and his/her agency will be reinforced by the self-monitoring and adherence to self-management recommendation. In such a way, the patient will be more prepared to participate in processes of clinical decision-making (40).

From the healthcare professional's perspective, bridging the gap between the hospital and patient's home is a criterion of quality of care, contributing to enhancing accessibility and care continuity. Among many other advantages, mHealth resources allow to reduce access and continuity inequities related to geographical location and financial constraints. They additionally allow safety of care and treatment along with their telemonitoring resources, with healthcare professionals having access to disease and illness indicators during patient's daily life functioning (17).

The integration of mHealth resources, and particularly the mHeart.4U, into CR will therefore make it possible to personalize the intervention parameters, facilitate monitoring and tracking according to patient's personal preferences and clinical needs, which is expected to improve the patient's recovery and health status (17). This flexibility aspect related to tailoring in mHealth programmes is a valuable feature compared to conventional programmes. Particularly in CR programmes, tailoring is likely to go beyond prompting adherence as the person closer relates to the intervention content and format, to embrace intervention effectiveness as specific e-management recommendations are determined upon the patient's clinical profile.

The success of mHeart.4U intervention will be a step toward the use of technological interfaces as an integral part of CR programs. Altogether, these programmes are likely to facilitate the management of resources for healthcare professionals and reduce inequality of access to healthcare for CVD patients. In the western world healthcare systems, the complementarity of mHealth solutions to the treatment, care and rehabilitation along the chronic diseases pathway is mandatory if aiming for sustainable, integrated healthcare systems that endorse patient's willingness to be part of their treatment journey, while assisting them at distance.

## Ethics Statement

The project will be reviewed by the Ethics Committee of both hospital units where the study will unfold. The study was registered in ClinicalTrials.gov on the 18th January 2022 with the number NCT05196802.

The study findings will be published in international peer-reviewed scientific journals in accordance with Consolidated Standards of Reporting Trials, including the extension for non-pharmacological treatment interventions (41). Dissemination through scientific meetings will also be conducted. Workshops will be organized to disseminate the results to healthcare professionals and other stakeholders. Furthermore, citizen-friendly reports will be elaborated to disseminate the results to the end-users in the society.

## Author Contributions

PS led the design of the study protocol and will coordinate the RCT. FV led the writing of the study protocol. SD contributed to elaborate the data analysis protocol and will assist in the statistical analysis of the RCT data. JM and LG provide advice on key study issues. PS, PF, and MA contributed to design the intervention. All authors contributed with important intellectual content to the study protocol and approved the final version for publication.

## Funding

This work was partially supported by the Center for Innovative Care and Health Technology (ciTechcare), Polytechnic of Leiria, Portugal. The work of FV was funded by the Portuguese Foundation for Science and Technology (FCT), CEECINST/00103/2018. The funder had no role in the clinical study protocol.

## Conflict of Interest

The authors declare that the research was conducted in the absence of any commercial or financial relationships that could be construed as a potential conflict of interest.

## Publisher's Note

All claims expressed in this article are solely those of the authors and do not necessarily represent those of their affiliated organizations, or those of the publisher, the editors and the reviewers. Any product that may be evaluated in this article, or claim that may be made by its manufacturer, is not guaranteed or endorsed by the publisher.
